# Environmental and public health co-benefits of consumer switches to immunity-supporting food

**DOI:** 10.1007/s13280-021-01693-w

**Published:** 2022-01-25

**Authors:** Ayesha I. T. Tulloch, Rachel R. Y. Oh, Danielle Gallegos

**Affiliations:** 1 School of Life and Environmental Sciences, University of Sydney, Sydney, NSW 2000 Australia; 2 School of Biology and Environmental Science, Queensland University of Technology, Brisbane, QLD 4000 Australia; 3 School of Biological Sciences, University of Queensland, Brisbane, QLD 4072 Australia; 4 German Centre for Integrative Biodiversity Research (iDiv) Halle-Jena-Leipzig, Leipzig, Germany; 5 Department of Ecosystem Services, Helmholtz-Centre for Environmental Research – UFZ, Leipzig, Germany; 6 Faculty of Health, Woolworths Centre for Childhood Nutrition Research, Queensland University of Technology (QUT), Brisbane, QLD 4101 Australia; 7 School of Exercise and Nutrition Sciences, Queensland University of Technology (QUT), Kelvin Grove, QLD 4059 Australia

**Keywords:** Behaviour change interventions, Biodiversity conservation, COVID-19, Health psychology, Sustainable food production and consumption

## Abstract

During COVID-19, there has been a surge in public interest for information on immunity-boosting foods. There is little scientific support for immunity-supporting properties of specific foods, but strong evidence for food choice impacts on other health outcomes (e.g. risk of non-communicable disease) and environmental sustainability. Here, we relate online recommendations for “immunity-boosting” foods across five continents to their environmental and human health impacts. More frequently recommended food items and groups are plant based and have lower land use and greenhouse gas emission impacts plus more positive health outcomes (reducing relative risks of mortality or chronic diet-related diseases) per serving of food. We identify trade-offs between environmental outcomes of increasing consumption of recommended food items, with aquatic environment impacts increasing with food recommendation frequency. People’s reliance on the Internet for health information creates an opportunity to consolidate behaviour change towards consuming foods with multiple co-benefits. Our study identifies win–win options for nudging online information-seeking behaviour towards more sustainable choices for terrestrial biodiversity conservation and human health.

**Supplementary Information:**

The online version contains supplementary material available at 10.1007/s13280-021-01693-w.

## Introduction

Biodiversity and environmental health are inseparable from people’s livelihoods and health (Frison et al. 2011; Jones 2017). There is increasing evidence that conserving biodiversity benefits human well-being through increasing household wealth and child health (Naidoo et al. 2019), restoring mental health (Ferraro et al. 2020), and reducing infectious disease (Rohr et al. 2020). However, campaigns designed to promote sustainable biodiversity-friendly behaviours are rarely framed in terms of human health, and strategies to improve public health are rarely applied to environmental behaviours (Nisbet and Gick 2008; Wahqvist and Gallegos 2020). As a result, conservation, health and nutrition organisations and government agencies have failed to deliver credible, consistent methods of incentivising consumers to switch to more biodiversity-friendly, healthy behaviours (Treves and Jones 2010). Biodiversity continues to decline at the highest rate in human history, and chronic diet-related diseases such as diabetes result in the deaths of millions of people each year (Martinez et al. 2020).

When people are provided with the right information, rewards and supportive economic and environmental conditions, interventions can enact lasting behavioural changes (Jepson et al. 2010; Gardner et al. 2014; Wood and Neal 2016). Information from people’s online social networks, social media and the Internet has the power to influence people’s behaviours, particularly in times of crisis (Brug et al. 2005; Maher et al. 2014). The coronavirus (COVID-19) pandemic has been accompanied by changes in food purchasing and consumption behaviours around the globe. Websites and social media have been flooded with articles and messages promising cure or prevention of COVID-19 infection. More than three quarters of these websites focus on diet as a way to boost immunity (Cassa Macedo et al. 2019). At the same time, there has been increased demand for healthier “immune-boosting” food (web searches for “immunity booster food” increased by 350% between 2019 and 2020; Rachul et al. 2020).

Although the only evidence-based approach to disease immunity is vaccination (Rappuoli et al. 2014), dietary patterns can affect people’s health and disease susceptibility (Yousafzai et al. 2013). Clinical studies show that poor nutrition increases the risk of infection, and that certain nutrients (e.g. vitamin D, zinc, phytonutrients) could improve defence function and resistance to infection (Wu et al. 2019). Evidence-based links between specific food items and immunity from transmissible disease are few (Jayawardena et al. 2020; Venter et al. 2020), and there is no evidence that any individual food provides enhanced “immune boosting” protection against COVID-19. However, the kinds of COVID-19 information to which people are exposed could influence short- and long-term health-related behaviours and attitudes (Blouin-Genest et al. 2020; Bursztyn et al. 2020), including switching from regular routines to new habits. If so, it is imperative that this information is accurate, and that the benefits of switching to new habits outweigh the risks. Possible risks of COVID-19 behaviour change include vaccine avoidance, toxic responses to food substances, malnutrition and decreased immunity (Naja and Hamadeh 2020). The current power and trust in online information (Cole et al. 2016) raises concern from the scientific community that dietary advice not backed by scientific evidence could lead to such negative outcomes. Nevertheless, public reliance on online information presents an opportunity for the science community to harness enthusiasm for behaviour change. This could be achieved by using people’s exposure to food immunity information as a pathway to building knowledge about benefits and risks of different food choices for public health and the environment.

Many foods marketed as “immunity boosting” are indeed healthy. However, not all food habits have positive health outcomes, and not all healthy habits are environmentally sustainable (Macdiarmid 2013; Dwivedi et al. 2017; Luis et al. 2018). Here, we address this trade-off by identifying opportunities in people’s exposure to dietary choice information that could consolidate positive behavioural changes to diets that are both healthier and more sustainable (Nisbet and Gick 2008; Hopkins et al. 2020). We explored online recommendations for food items purported to have immune-boosting properties across six worldwide case study regions, to discover potential dietary choices that the public is exposed to. We quantified the reporting rates of individual food items through a content analysis of 150 webpages that would be discoverable to the public if they searched for the term “food immunity” in six predominantly English-speaking nations (USA, Australia, UK, Singapore, Nigeria and India) on five continents. We then calculated the likely impacts of producing each proposed “immunity-boosting” food on the environment, and the likely impacts of eating the food on consumer health. We used this combined dataset to assess whether food items with high online reporting rates, would, if included in diets, have better outcomes for human health and environmental sustainability than food items with low visibility on webpages recommending immunity food.

We assessed possible effects of proposed “immunity-boosting” food items on people versus the environment using established globally applied indicators. For health, we evaluated five standard health risk indicators: all-cause mortality (ACM), coronary heart disease (CHD), colorectal cancer (CRC), diabetes and stroke (Clark and Tilman 2017). For the environment, we evaluated five standard environmental risk indicators: land use (a surrogate for the impacts of habitat loss on terrestrial biodiversity), greenhouse gas (GHG) emissions (a surrogate for the impacts of climate warming on biodiversity), freshwater withdrawals weighted by local water scarcity (a surrogate for the impacts of water use), and two forms of nutrient pollution—acidification (lowering of soil pH caused by atmospheric deposition of chemicals) and eutrophication (release of nutrients such as nitrogen and phosphorus into waterways or groundwater) (Poore and Nemecek 2018b). We selected these health and environmental indicators because plausible causal metabolic mechanisms between food consumption and health outcomes exist for these foods and because the health and environmental impacts of these foods are well documented through meta-analyses (Supplementary Data S1). For all proposed “immunity-boosting” food items, we evaluated the likely health and environmental outcomes of behaviour changes by consumers who took the advice of webpages and consumed an additional serving of each recommended food item per day.

## Materials and methods

### Frequency of food recommendation

We performed a general search for the term “immunity food” in Google.com from Brisbane, Australia in July 2020. Google® was chosen as a search engine was because it has over 90% of the search engine market share (Statista 2019). Prior to conducting the search using the browser Google Chrome®, we deleted all cookies and browser history to limit personalisation of the search results returned and maximise the global reach of the web pages returned (Cassa Macedo et al. 2019), although geolocalisation could not be prevented as this is linked to the IP address used for the connection to the Internet. We transferred the first 25 uniform resource locators (URLs) in the search engine result page (SERP) to a spreadsheet. We then repeated this search process for five additional English-speaking regions of the world, to capture a range of consumer markets reflecting variations in social, cultural and geographical environments that influenced diet, food availability and food choices. These regions were India, Singapore, Nigeria, the UK and the USA and were achieved through adjusting the “search region” settings in Google® to India, Singapore, Nigeria, the UK and the USA.

To be included in the final review, the webpage had to (1) provide specific recommendations linking dietary choices to immunity (e.g. “Top 8 foods to boost your immunity”), and report at least one food item as having immune-boosting properties; (2) be freely accessible, without requiring registration or paywall to access them; and (3) have minimal advertisements or marketing for a particular product or brand. The webpage could include recipes but could not consist entirely of recipes.

For each webpage, we performed a content analysis by identifying all food items that were named in the text and suggested by the webpage as having beneficial effects for immunity. Food items that were presented as either as a photo or drawing (instead of in text) but clearly identifiable, were also included in our identification process (Cassa Macedo et al. 2019). We excluded generic food groupings such as “meat” and “vegetables”. From performing this content analysis across 150 webpages, we arrived at a final list of 183 food items, which were then allocated to one of 25 broad food groups. Food groups were determined based on published groupings of food nutritional quality in the health literature. We chose to assign each food item of one of the 25 broad food groups because food items are typically assessed together in public health and disease studies due to similarities in nutritional outcomes, composition of key minerals, vitamins and proteins (e.g. vitamin-C rich fruit and vegetables) (Aune et al. 2017; Clark et al. 2019). Aggregating the foods in this way also deals with recommendation bias resulting from cultural dietary differences. For instance, certain food items from the same food group were specific to particular regions (e.g. Indian gooseberries were only recommended on Indian webpages, blackcurrants were specific to UK webpages), whereas others were recommended across all six regions (e.g. blueberries, strawberries). Of the identified food items, 33 food items and 3 food groups were excluded from subsequent analyses, as meta-analyses of health and environmental impacts were not available for them and therefore dose–response curves could not be calculated. Specifically, these were predominantly food additives that do not represent a large proportion of a daily diet (i.e. herbs, spices, seeds, food additives, supplements, and medicinal products), and there was no data on the relative risk (RR) of environmental and health impacts, because of the challenges in determining appropriate serving sizes for such food items (e.g. the recommended dose for seeds and other herbs and spices ranges from 1.5 g/day for caraway and cardamom seeds to 20 g/day for dried onion) (Kaefer and Milner 2008).

### Food impacts on health and environment

The final dataset for modelling of environmental and health impacts of foods included 150 web pages (25 from each region) and 150 food items from 23 food groups. We matched each food item to the results of recent meta-analyses from the health literature assessing the RR associated with consuming different foods (Aune et al. 2017; Clark and Tilman 2017) and did the same for recent meta-analyses of the environmental impacts of food item production (Clark and Tilman 2017; Poore and Nemecek 2018a, b). The health outcomes we evaluated are the RRs of disease resulting from consuming an additional serving of a food per day relative to the average intake of that food observed in a cohort study. If RR > 1, consumption of an additional serving is associated with increased disease risk compared to the average risk of that disease, and if RR < 1, this consumption is associated with decreased disease risk. The food-dependent health data are derived from 50 dose–response meta-analyses and a recent global review (Supplementary Data S1). The five environmental outcomes we evaluated are the impacts of production of each food per unit of primary nutritional benefit (also known as a functional unit, e.g. 1 kg of brown rice or 1 l of milk), as estimated by meta-analyses of life-cycle assessments (LCAs) that account for the environmental impacts of plant and animal production (Poore and Nemecek 2018b) (Supplementary Data S2). The selected impacts were those that might directly affect biodiversity during agricultural production of the food item, including the production, manufacture, and use of agricultural inputs, seed, equipment, and cropland (Poore and Nemecek 2018b), and excluded post-farm processes (transport, processing, retail, food preparation and losses) that have indirect impacts on biodiversity.

#### Health impacts of food

We matched each food item (identified through the webpage search) to the RR associated with consuming them. RR was derived from meta-analyses from the health literature (Aune et al. 2017; Clark and Tilman 2017). For each food item, we first analysed the impact on adult health from consuming an additional serving of food per day, as reported from prospective cohort studies in the scientific literature. Prospective cohort studies follow populations through time to examine the health outcomes of changes in risk factors, such as consumption of different foods or dietary patterns. Dose–response analyses report the health impact of consuming a serving of food per day (1 serving more than the cohort average), for example, the health impact of consuming an additional serving of red meat per day. We used dose–response meta-analyses in this analysis for several reasons. First, in comparison with other forms of prospective cohort studies (e.g. quintile analyses or substitution analyses), dose–response meta-analyses have been conducted for most commonly consumed food groups. Second, dose–response studies allow for more direct comparison of the health and environmental outcomes of different foods in quantities that might be consumed at a single meal (Clark and Tilman 2017), compared with quintile and substitution analyses that are difficult to standardise across different indicators. For example, quintile analyses report the health outcome of the subgroup that consumes the least red meat against the health outcome of the subgroup that consumes the most red meat, but the amount of meat consumed might vary between studies. Studies examining food substitution report the health outcome of substituting one food for another, for example, the health outcome of substituting one serving of red meat per day with an equivalent amount of chicken per day, and are useful for evaluating the health impacts of particular product substitutions (e.g. sugar substitutes) but less useful for investigating how increasing or decreasing consumption of a particular food might impact health and the environment.

We synthesised results from 50 recent dose–response meta-analyses and a recent global synthesis of the health impacts from selected food items (Clark et al. 2019) to determine how five health outcomes were impacted by consuming an additional serving of each type of food per day (see Supplementary Data S1 for the dose–response meta-analyses included in this analysis). We used meta-analyses of dose–response studies as this is more reliable and reflective of the broad magnitudes of health impacts of different foods than individual dose–response studies, whose results may vary across cohorts and study methodologies. We collated data on the health impacts of consuming an additional serving of food per day for 41 food items or groups (see Supplementary Data S1)—these were aggregated to 23 groups that were broadly similar in nutritional benefits and impacts.

The selected health outcomes were four indicators of chronic disease incidence—CHD, CRC, type II diabetes, and stroke—and one indicator of mortality risk (ACM, Supplementary Table S1). The existence of dose–response relationships from multiple cohorts, together with plausible pathways that explain the change in disease risk, suggests that the risk relationships are reflective of biological processes and are broadly applicable. We also calculated two aggregate risk indicators: the average relative health impact (the average of the relative impacts for all five health outcomes) and average relative chronic disease impact (the average of the relative impacts for CHD, CRC, type II diabetes, and stroke).

The serving sizes used in this analysis are 200 g for sugar-sweetened beverages (SSBs); 200 g for milk and yoghurt; 50 g for cheese; 100 g for yoghurt and kefir (fermented dairy products) (Aune et al. 2013); 150 g for potatoes; 100 g for chicken, red meat, fish, fruits, and vegetables; 50 g for processed red meat, eggs, and legumes; 30 g for refined grains and whole grain cereals; 28 g for nuts (Blomhoff et al. 2006; Aune et al. 2016a); 20 g for saturated and unsaturated fats and oils; 20 g for chocolate; and 2 cups (approx. 20 g) for other stimulants (tea and coffee). In cases where dose–response meta-analyses reported health outcomes at different serving sizes, we calculated the reported RR of disease per serving. For food items without specific meta-analyses of dose–response analyses but with data available for similar food items within their food group, we calculated the average impact for the health indicator across their food group and allocated this value as their health impact.

#### Environmental impacts of food

We determined, for each of the 150 food items, how agricultural production of a serving of each food impacted five types of environmental degradation—GHG emissions, land use, scarcity-weighted water use, and acidification of soil and eutrophication (two forms of nutrient pollution)—using data from two global LCA systematic reviews and meta-analyses (Poore and Nemecek 2018a, b; Clark et al. 2019). LCA is a standardised method to estimate the environmental impacts per unit of food production. While data from life-cycle meta-analyses (LCA) are primarily from high-income and high-input nations, studies using different methods of environmental assessments have shown that the relative rankings of environmental impacts of different foods are similar across regions even when the environmental impacts of food production per unit of food produced vary across regions (Herrero et al. 2013; Carlson et al. 2017; Poore and Nemecek 2018b).

As for disease risk, using meta-analyses of LCAs can be considered more reliable and reflective of the general magnitudes of environmental impacts of different foods than individual LCAs because of potential variation between individual LCAs. The global reviews of LCAs from which we obtained estimates of GHG emissions, land use, nutrient runoff (specifically eutrophication) per gram of food (Poore and Nemecek 2018a), estimated environmental impacts from cradle to consumption, and weighted impacts per unit of food production nationally and internationally to be representative of current global average production location and methodology. This system boundary accounts for all impacts that occur from pre-farm and on-farm activities such as fertiliser production and application, infrastructure construction, and on-farm fossil fuel, as well as post-farm activities such as transportation, processing, refrigeration, and cooking. GHG emissions from land-use change are also included in these estimates. Using impacts weighted by current global production methodologies accounts for differing environmental impacts of producing a given food across geographical regions and production methodologies (Herrero et al. 2013; Clark and Tilman 2017) and reduces the risk of any potential data biases resulting from using environmental impacts data biased towards a single production location or methodology.

To better allow broad comparisons between the overarching health and environmental impact of different foods, we followed the methods of Clark et al. (2019) to recalculate the environmental impact of each food as its relative environmental risk by first calculating the impact of producing a food for each indicator relative to the impact of producing a serving of mushrooms. We calculated three aggregate risk indicators: the average relative environmental impact (the average of the relative impacts for all five environmental outcomes examined here), average relative terrestrial biodiversity impact (the average of the relative impacts for GHG emissions, land use and acidification of soil), and the average relative aquatic biodiversity impact (the average of the relative impacts for scarcity-weighted water use, acidification of soil and eutrophication). Acidification of soil was included in both land and aquatic impacts as soil acidification directly impacts surface and groundwater quality as well as water-dependent systems. As such, a food group with an averaged relative environmental impact of three indicates that producing a serving of that food group results in, on average, three times the environmental impacts across the five environmental outcomes examined here than does producing a serving of mushrooms. For food items without specific LCA studies, we calculated the average environmental impact for each environmental risk indicator across their food group and allocated this as the environmental impact for that individual food item.

### Predicting frequency of recommendation from impacts

All analyses were conducted in the statistical programming environment R version 3.6.2 (R Development Core Team 2019). We first assessed variations in the recommendation frequency of each food item across the six geographical regions and 23 nutritional food groupings. We then identified human health and environmental outcome predictors associated with food dietary choices by building two sets of mixed-effects models using the lme4 package (Bates et al. 2015)—one relating health and environmental outcomes to food-group recommendation frequency, and one for individual food items.

The first model set fit a mixed-effects model with a Poisson link where the response variable was the frequency of recommendation of each of the 23 food groups across the 25 webpages evaluated for each of the six regions (an integer taking possible values of 0 to 25, *n* = 132). The predictor variables were plant/animal food-group classifier (binary), food-group impact (see Supplementary Table S1 for a description of each of the 15 impact indicators), and an interaction term between the plant/animal classifier and food-group impact (Supplementary Table S2). We had a total of 15 models—one for each of the 10 individual health and environment indicators (ACM, CHD, CRC, Diabetes, Stroke, Land Use, GHG, AP, EP, Water Use) and five aggregate indicators: RRAll (average across all 5 health indicators); RRDisease (average across chronic disease indicators); EnvAll (average across all environmental indicators); EnvTerr (average across all indicators of terrestrial impacts—Land Use, GHG, AP); and EnvAq (average across all indicators of aquatic impacts—AP, EP, Water Use). We included the interaction term as previous studies have found significant differences in impacts between plant and animal food groups (Clark and Tilman 2017), and these differences could influence how food groups are recommended. We specified geographical region as a random slope to account for possible cultural variation between regions in the types of food groups that are recommended.

The second model set fits a mixed-effects model with a binomial link to food item recommendation frequency and related this frequency to the average impact of all food items for each of the 15 health and environmental indicators. The response variable was the occurrence of each of the 150 food items across the 25 webpages evaluated for each of the 6 regions (a binary value of 0 or 1, *n* = 22 200). The predictor variables were a binary plant/animal food-group classifier and food-group impact (1 of each of the 15 impact indicators). An interaction term between the plant/animal classifier and food item impact was included. Interactions within food groups were not included as some food groups were singular in either or both environmental or health impacts (e.g. mushrooms, butter). We specified geographical region and study as random slopes to account for variation between regions and studies in the food items that are recommended.

For all models, continuous environmental and health indicators were scaled as certain food items, and food groups create high skew in the data due to having impacts an order of magnitude higher from the majority of food items (Poore and Nemecek 2018b; Clark et al. 2019). Quantile plots, histograms of residuals, and plots of residuals against fitted values were also examined for non-normality, heteroscedasticity, or presence of outliers. Significant variables were identified (*p*-value < 0.05) using the anova() function from the lmerTest package (Kuznetsova et al. 2017).

### Evaluating variability in sets of food recommendations

We conducted multivariate analysis (principal components analysis, PCA) to evaluate similarities in the composition of the set of food-group recommendations within each webpage in terms of (a) the occurrence of each food group within the set of recommended foods, and (b) the average impact (for each indicator) from the set of foods recommended on that website. We used the FactoMineR package in R (Lê et al. 2008) to run each PCA and evaluated overlap between either regions or webpage governance/goals. For the food-group occurrence PCA, the data on food-group recommendations were converted into a presence–absence matrix of webpage by food group (dimensions 150 × 23), with a 0 indicating that the food group was not recommended by that web page and a 1 indicating that it was recommended. For the average impact PCA, we first calculated the average impact for each of the ten indicators of the foods recommended in the webpage, resulting in a matrix of webpage by average indicator impact (dimensions 150 × 10), and containing continuous values from 0 to the maximum impact (124.3). To explore differences between regions in recommendations and their potential environmental and health impacts, we grouped studies according to the country to which the website belonged when evaluating individual contributions of each study to the total variance. To explore differences between broad webpage types in recommendations and impacts, we categorised each webpage into six types by its broad governance/goals: Blog/Lifestyle site (*n* = 26), Commercial site (product sales, *n* = 26), Education (with oversight from a medical authority, *n* = 18), Health care/Insurance (*n* = 16), Media (magazine/news/TV/radio, *n* = 60), and Other (restaurant/travel guides, public forums, *n* = 4). After plotting the PCA for each analysis, we drew ellipses to cluster points by either region or webpage type, to enable visualisation of recommendation or impact overlaps between groupings.

We also calculated the Bray–Curtis dissimilarity indices (Bray and Curtis 1957) between the set of food recommendations for each region’s websites to assess concordance between the recommended sets of food groups in each website. A low Bray–Curtis dissimilarity index between a pair of websites indicates that the food groups recommended on each website were highly similar. Finally, we used ANOSIM in R (vegan package) to test whether there was a statistical difference in food-group recommendations or the average impacts of food-group recommendations between either countries or webpage types.

## Results

### Frequency of food recommendation

**Fig. 1:**
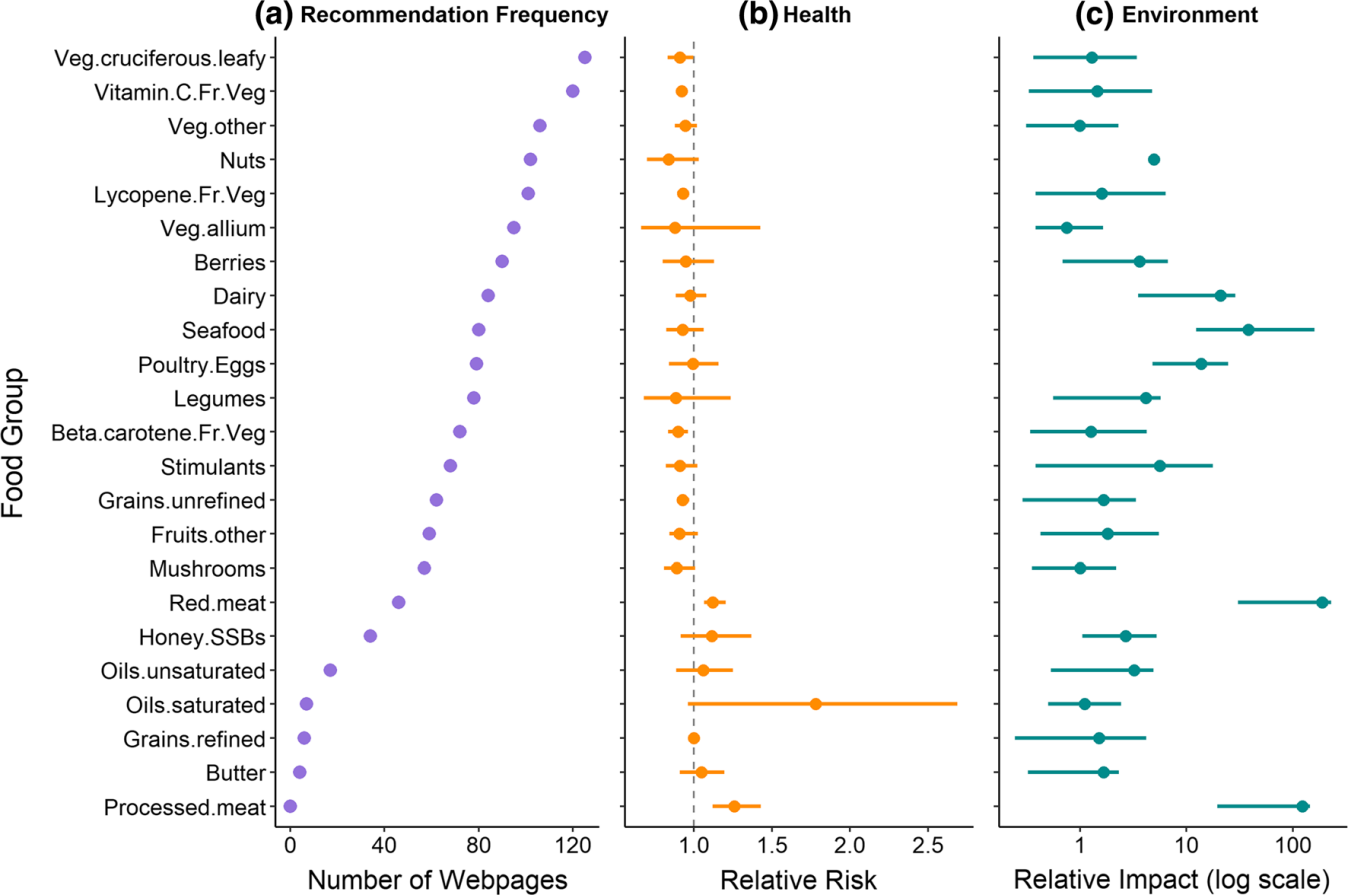
The 150 recommended food items grouped by 23 major nutritional food groups and ranked in order of frequency of recommendation on webpages offering advice about immunity-boosting foods, showing **a** total number of webpages that recommended at least one item from each food group, **b** mean health impacts with 95% confidence intervals (averaged across five indicators of RR of mortality or disease), and **c** mean environmental impacts with 95% confidence intervals (averaged across five indicators of land and water use, greenhouse gas emissions, and nutrient pollution). Dotted line in **b** indicates switch in health risk from reduced risks of disease and mortality (left of line) to increased health risks (right of line)

Across all 150 webpages and 6 regions, 83% of the 2556 recommendations for food items that might “boost” immunity were for plant-based foods, with little variation between countries in the relative number of plant-food recommendations (from a low of 77.7% in the UK to a high of 85.2% in Australia). The top six most frequently recommended food groups were plant based: cruciferous and leafy green vegetables (e.g. broccoli), vitamin-C-rich fruit and vegetables (e.g. citrus fruits, pineapple), other vegetables (e.g. root vegetables), nuts, lycopene-rich fruit and vegetables (e.g. tomatoes, red peppers/capsicum), and allium vegetables (e.g. onions, garlic) (Fig. 1a). Increasing the daily dose of any food item within these six most frequently recommended food groups by one serving will, on average, reduce the risk of ACM and chronic diseases (Fig. 1b; Supplementary Fig. S3). The average environmental impacts of these top-ranked food groups are also generally lower than rarely recommended food groups (Fig. 1c; Supplementary Fig. S4).

**Fig. 2:**
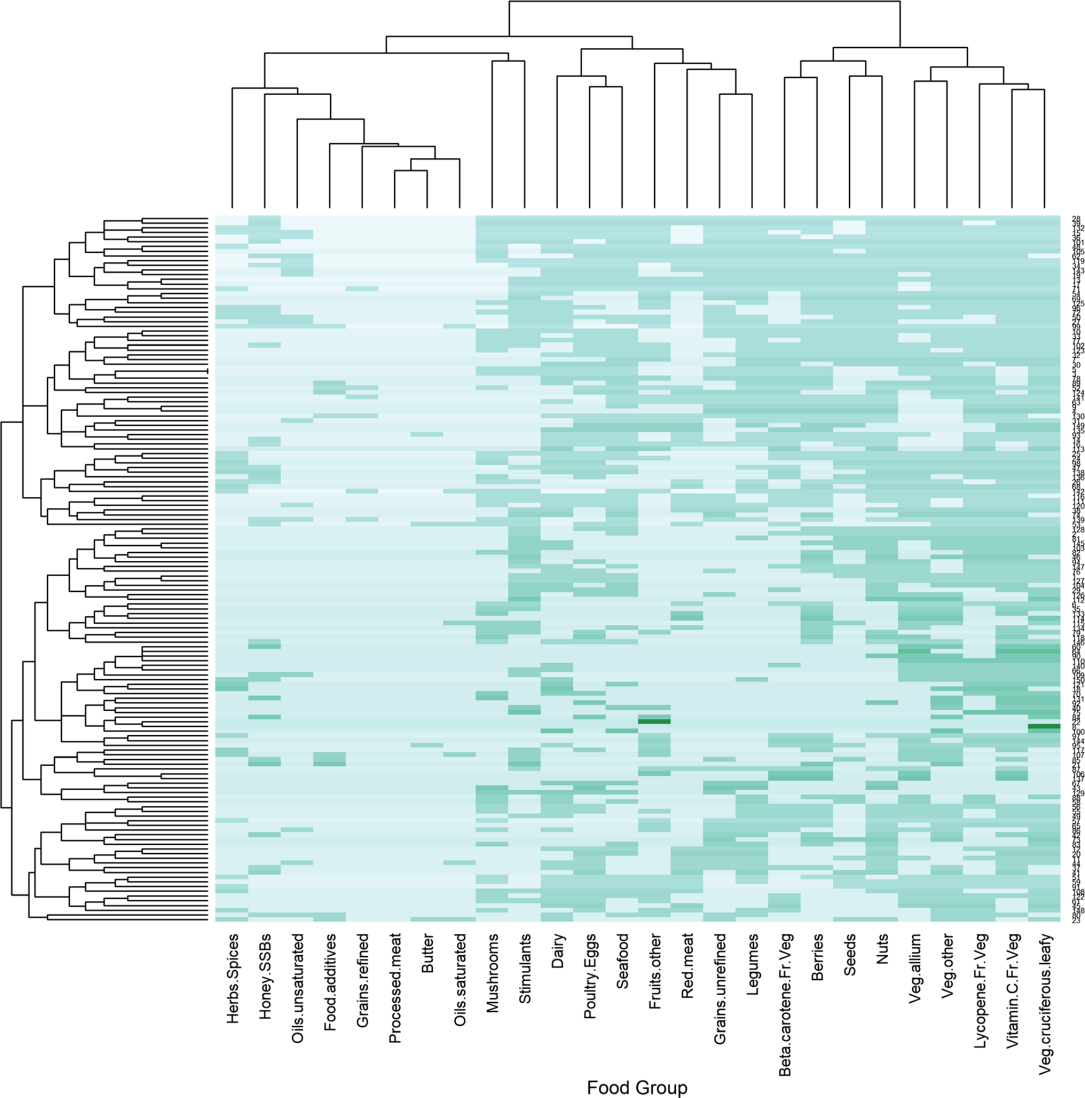
Heatmap of the entire set of recommended food groups from 150 webpages across 6 regions offering advice about food items purported to have “immunity-boosting” properties. Food group frequencies and webpage composition (food groups recommended by each webpage) are clustered by the top dendrogram, and webpages clustered by the side dendrogram, using hierarchical clustering by Bray–Curtis distance

Individual webpages differed in the food items that they recommended (mean Bray–Curtis dissimilarity across all webpages 47.7 ± 18.4% standard deviation). We identified certain areas of overlap in recommendations using a heat map (Fig. 2). Websites that recommended red meat were more likely to recommend other animal products (e.g. seafood, poultry, dairy) than those recommending vegetables and nuts (Fig. 2). Websites recommending food items with negative health outcomes (e.g. sugar, fats, oils, refined grains) were less likely to recommend plant foods with positive health outcomes such as vegetables, legumes, and nuts (Figs. 1, 2; Supplementary Fig. S2).

Regions had high overlap in food-group recommendations despite within-region variation in the composition of webpage recommendations (Supplementary Figs. S1, S2a, b). Some food groups were, however, more frequently recommended by certain regions than others (Supplementary Fig. S1, Supplementary Data S3). For example, two thirds of all recommendations for refined grains came from the UK webpages, half of all recommendations for saturated oils were from Indian webpages, and most sugar-based food recommendations were from US (30%) and Australian (24%) webpages. Dairy and poultry products were less frequently recommended in Australia, while red meat was least often recommended in India and Singapore (Supplementary Fig. S1).

We also found no significant differences between webpage types in the composition of food-group recommendations (Supplementary Fig. S2c). All webpage types recommended cruciferous vegetables the most frequently and recommended oils, butter, and refined grains least frequently. We did observe differing patterns in the relative frequency of certain food-group recommendations between webpage categories. Sites representing educational organisations (e.g. not-for-profits, universities) with medical oversight had similar relative recommendation frequencies of cruciferous vegetables to blogging/lifestyle sites (10.5% of all recommendations in this category), which was lower than for other sites (up to 15.2% of recommendations for health care/insurance sites; Supplementary Fig. S1b). Sites focused on commercial product sales recommended beta-carotene-rich fruits and vegetables two to four times less than other sites, had the highest relative recommendation frequencies for berries, vitamin-C-rich fruits and vegetables and stimulants, and had the lowest recommendation frequencies for unrefined grains and legumes compared with other webpage categories (Supplementary Data S3). Health care and insurance providers, on the other hand, had the lowest relative recommendation frequencies for stimulants and berries, and the highest recommendation frequencies for dairy, poultry, and eggs and red meat (Supplementary Fig. S1b). Sites representing news media and magazines had the highest recommendation frequencies of nuts and lowest recommendation frequencies of vitamin-C-rich fruits and vegetables.

### Impacts of consuming recommended foods on health and environment

**Fig. 3:**
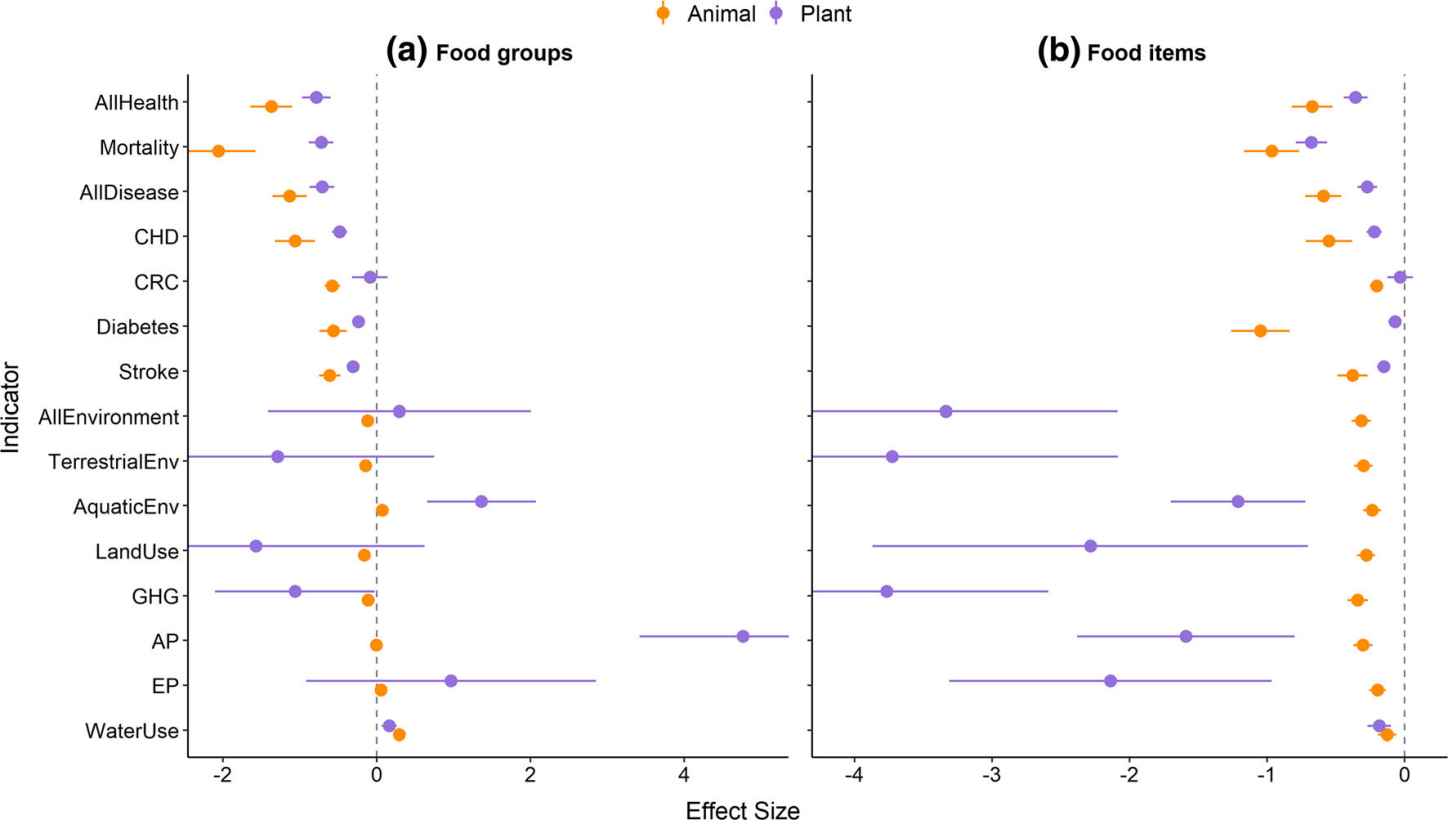
Mean conditional slope estimates (± 95% CI) for 15 health and environmental impact indicators, from mixed-effect regression models explaining the relationship between **a** 23 food-group recommendation frequencies and their health or environmental impacts, and **b** 150 food item recommendation frequencies and their health or environmental impacts. More negative effect sizes (i.e. < 0) indicate lower averaged impacts on health or environment with increasing recommendation frequency, and more positive effect sizes indicate higher (i.e. worsening) impacts on health or environment. Slopes are estimated from the model curve when relative risk = 1 for health impacts and average indicator value = 1 for environmental impacts

**Fig. 4:**
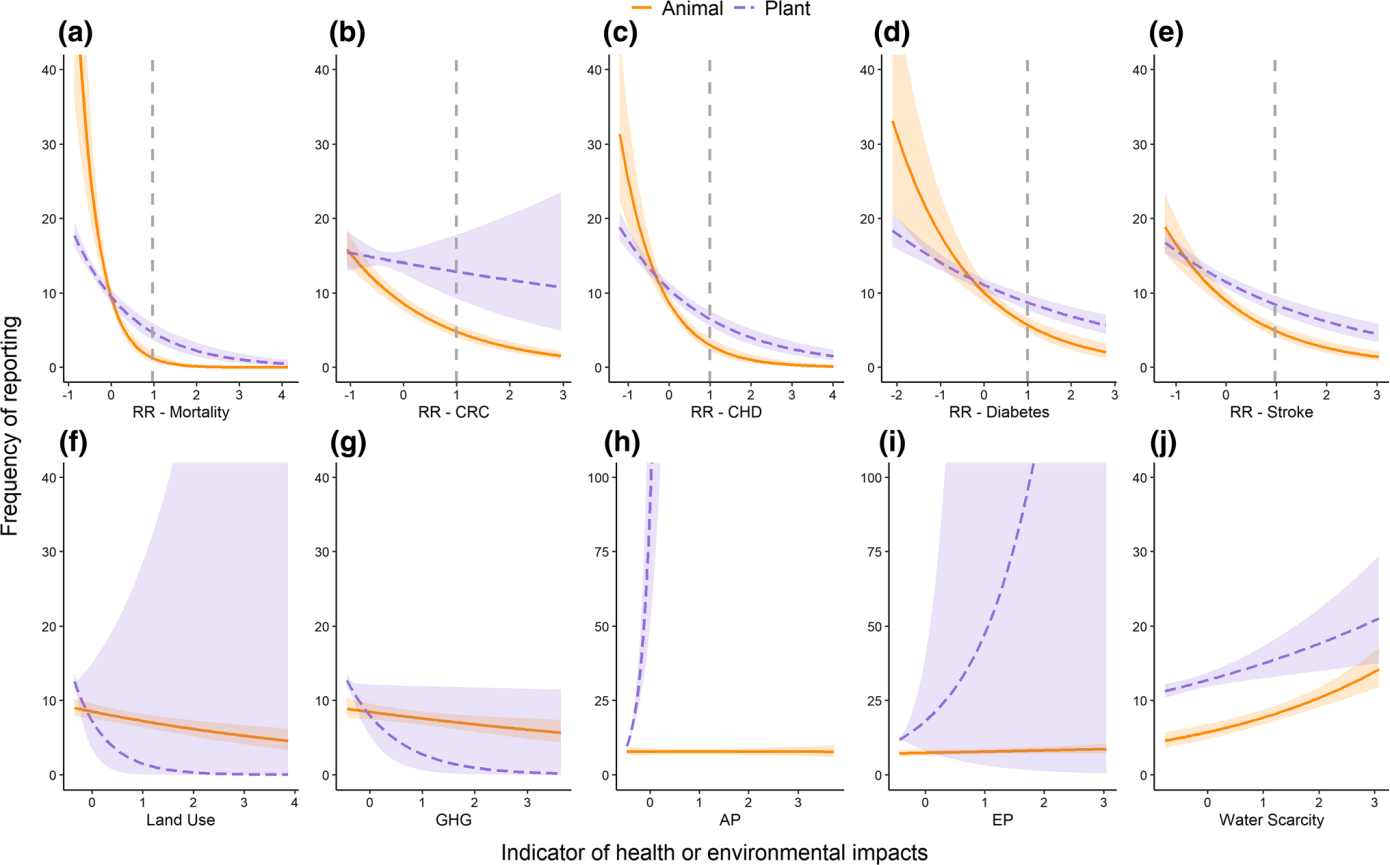
Interaction plots from mixed-effect regression models investigating the relationship between food-group recommendation frequency and **a** all-cause human mortality, **b**–**e** human chronic disease risk, **f** land use, **g** GHG emissions, **h**, **i** nutrient pollution and **j** water use. Relationships for animal food groups (e.g. poultry, dairy; orange) and plant food groups (e.g vegetables, nuts) shown separately. Dotted line in **a**–**e** indicates switch in health risk from reduced risks of disease and mortality (left of line) to increased health risks (right of line). Shaded areas represent 95% confidence intervals

Regardless of whether food groups were animal or plant based, our models showed that food groups with higher online recommendation frequencies had significantly lower relative health risks than food groups with lower recommendation frequencies. As food-group recommendation frequency declined, the RRs of ACM and chronic diseases increased (Figs. 3a, 4a–e). The one exception was CRC—while animal food groups with high recommendation frequency had a significantly lower RR of CRC, there was no significant relationship between the risk of CRC and plant-food-group recommendation frequency (Fig. 4b).

Highly recommended food groups tended to have lower impacts on terrestrial environments compared with less-recommended food groups. These impacts were significantly lower for GHG emissions for both plant and animal food groups, as well as for land use and the aggregated terrestrial impacts indicator for animal food groups (Figs. 3a, 4f, g). In contrast, highly recommended food groups tended to have higher impacts on aquatic environments, especially for plant food groups (Figs. 3a, 4h–j).

When we disaggregated food groups to investigate patterns for individual food items, regardless of whether food was animal or plant based, we found similar patterns. Consumption of an extra dose of a more frequently recommended food item over a less-recommended food item, resulted in lower environmental impacts and health risks (Fig. 3b). However, not all recommended food items have positive co-benefits for reducing the risks of mortality, disease and environmental impacts. Yoghurt, eggs and poultry (predominantly chicken) comprised the 6th, 11th and 17th most frequently recommended food items, despite being in the top quartile (i.e. the worst outcomes) for averaged environmental and health impacts.

Although there was a clear trend in increasing impacts on health and environment with increasing food-group recommendation frequency, the slope of this relationship varied depending on whether foods were plant or animal based (Figs. 3, 4). For health impacts, animal-based food groups showed a steeper decline than plant-based food groups (Fig. 4a–e). The relationships between health outcomes and food-group recommendation frequency were less pronounced for plant-based groups because increasing the daily dose of most plant-based food groups generally results in reduced health risks (Aune et al. 2016b, 2017; Clark et al. 2019; Hemler and Hu 2019), regardless of which food group the plant belongs to (Fig. 3a, b; Supplementary Data S4 and S5, Supplementary Fig. S3). Only animal food groups with very high recommendation frequencies (dairy, seafood, poultry/eggs; Fig. 1a) were associated with a reduced risk of chronic disease or mortality on average (Supplementary Fig. S3). Animal-based food groups with low recommendation frequency (e.g. butter, processed and unprocessed red meat) had increased risks of chronic disease or mortality (Figs. 1b, 4a–e). The recommendation frequency of these animal-based food groups with poor health outcomes was also lower than for plant-based food groups with low health outcomes (Fig. 4a–e).

In contrast with health impacts, the recommendation frequency of plant-based food groups declined faster than for animal-based food groups as land use and GHG impacts increased (Fig. 4f, g). This indicates that there were higher benefits for terrestrial environments from eating the most frequently recommended plant groups compared with those that were rarely recommended.

The relationships between aquatic impacts of food production and food-group recommendation frequency were inconsistent with the overall positive finding for other health and environmental indicators of higher recommendation frequency being associated with less severe impacts. For freshwater scarcity, the relationship was the converse of what we found for health, land, and emissions impacts. As recommendation frequency increased, so did freshwater impacts, regardless of whether foods were plant or animal based (Fig. 4j). The significant increase in freshwater impacts with food-group recommendation frequency was driven primarily by high water use from producing dairy, farmed seafood, nuts, some fruits and legumes (Poore and Nemecek 2018b; Clark et al. 2019) (Supplementary Fig. S4). For nutrient pollution (Fig. 4h, i), the relationship with food recommendation frequency differed depending on whether foods were plant or animal based (Fig. 4h, i). There was no relationship between acidification or eutrophication impacts and food-group recommendation frequency for animal food, whereas impacts for both nutrient pollution indicators increased with food-group recommendation frequency for plant food (Figs. 3a, 4h, i). The divergence in results for land versus water impacts is influenced by high aquatic environment impacts from producing food groups such as nuts, berries and some vegetables. These foods were highly recommended across all regions but result in relatively higher eutrophication and acidification impacts compared with less-recommended food groups such as grains and oils (Supplementary Data S4 and S5, Supplementary Fig. S4).

We found no significant differences between regions or between webpage types in the impacts of food-group recommendations (Supplementary Fig. S2b, d). Principle component analysis showed that all regions had high overlap in the average impacts of their webpage food recommendations (Supplementary Fig. S2b), and all webpage governance/goal types overlapped in impacts (Supplementary Fig. S2d).

## Discussion

There are many foods that, when taken as part of a diverse, balanced diet, are genuinely beneficial for health and environmental sustainability (Clark et al. 2019). However, providing easily accessible, verifiable information for consumers on the impacts of food choices is challenging, making sustainable, well-informed food choices difficult. Our study obtained a global overview of the information that the public is exposed to about dietary food choices when they are interested in improving their health outcomes in relation to immunity to a communicable disease.

We found that the food items recommended on webpages focused on immunity advice have the potential to reorient the food system to a more sustainable environmental trajectory, while delivering health benefits to consumers. Highly recommended plant and animal food items were associated with significantly improved health outcomes in terms of reduced risk of chronic disease and mortality, with plant-based food items recommended the most in the context of improving immunity (Fig. 1). The only health indicator analysis that did not show significant increasing health benefits with increasing recommendation frequency was CRC in association with plant-based foods. This is because there is relatively little difference in CRC risk between plant-based dietary food groups, and low correlation between the RRs of CRC and other diet-related diseases such as CHD and diabetes (Clark et al. 2019). In addition to health benefits received by consumers, we also found that increasing consumption of the most highly recommended plant-based food items by one serving per day could also deliver benefits for environmental sustainability. Foods that were recommended the most online had lower impacts on the terrestrial environment—specifically, lower GHG emissions and a lower total area of land used for farming (Fig. 3). This aligns with other studies reporting reduced environmental impacts associated with plant-based dietary shifts (Hallström et al. 2015; Mertens et al. 2017; Rohmer et al. 2018).

Not all highly recommended foods on webpages offering advice on dietary choices to boost immunity were beneficial to the environment or people’s health. We discovered trade-offs between environmental indicators wherein more recommended plant-based foods were correlated with less destructive land and climate change impacts but more degrading aquatic impacts (Fig. 4). This is likely due to higher variation within and between food production systems (e.g. organic vs. inorganic, and irrigated vs. rain fed) in the aquatic impacts of production compared with land and emissions impacts (Poore and Nemecek 2018b; Clark et al. 2019). This variability has led to global meta-analyses finding no significant difference between foods in overall aquatic impacts (Poore and Nemecek 2018b; Clark et al. 2019), even for foods that differ substantially for other environmental impacts (e.g. GHG emissions). As a result, there is low correlation between the impacts of food on water and on land (Poore and Nemecek 2018b; Clark et al. 2019). Foods with low terrestrial impacts could still have high aggregated environmental impacts if production is dominated by water-, fertiliser-, and pesticide-intensive methods that result in high acidification, eutrophication and water use. Variation in water impacts of individual foods offers an opportunity to build markets for more sustainable products by helping consumers understand which products have the lowest impacts across land and water, in comparison with many current initiatives (e.g. “Carbon Footprint”, “Rainforest Alliance”) that tend to focus only on land-based impacts (Grunert et al. 2014).

By exposing the public to information on the additional benefits of choosing foods with immune-supporting properties, webpages such as those reviewed here offer a potential pathway to building people’s knowledge about the benefits and risks of certain food choices for health and the environment. Many societies, particularly industrial agro-food economies, are increasingly distanced both geographically and socially from the source of their food, and therefore, from the impacts of their food choices on ecosystems and species (Clapp 2015; Mincyte and Dobernig 2016). This, combined with globalised industrialisation of food supply, has led to increasing homogenisation of food consumption worldwide (Brunelle et al. 2014). This was reflected in our results where the recommended food items had high overlap (Supplementary Fig. S2), despite coming from six culturally different regions. There has been recent attention on the possibilities of achieving co-benefits for human health from changes to global emissions policy, as climate policies targeting on emissions reduction should also improve air quality and reduce the risks of some illnesses (West 2013). Our results suggest that multiple benefits could also be achieved through consumer-driven changes to food purchasing and consumption behaviours related to maintaining health during a global pandemic. Growing reliance on the online informational environment (the “infosphere”) to inform and influence personal choices and decisions (Cassa Macedo et al. 2019) may present an opportunity to influence consumer behaviours towards more sustainable choices – even a one serve increase in vegetable consumption will have significant health benefits and reduce health systems costs (Cugelman et al. 2011; Guthrie et al. 2015; Lehner et al. 2016; Ekwaru et al. 2017; Byerly et al. 2018). This could be particularly effective in urban areas where the highest Internet use and the greatest food footprints occur (Hale et al. 2010; Ivanova et al. 2016), and during crises when peoples’ Internet use increases and when, in some situations, audiences perceive some online information to be more credible than traditional “news” outlets (Procopio and Procopio 2007).

While we found that the highly recommended food items in our analysis delivered both health and environmental benefits at a global scale, we did not evaluate local-scale impacts of foods and their supply chains. Differences in the availability of different food items in a person’s local environment will influence individual health outcomes and impacts of food choices on biodiversity by impeding or enabling access to certain foods. For example, some urban food environments have an abundance of (i.e. high accessibility to) low-cost unhealthy ultra-processed foods (Freudenberg and Galea 2008) and fewer less processed healthier food options (Horowitz et al. 2004; Moore and Roux 2006). In addition, some low-middle income countries (such as India and Nigeria) have greater exposure to globalised multi-national food companies, who are able to flood markets with ultra-processed and imported foods through liberalised trade agreements (Schram et al. 2018). Accounting for local characteristics pertaining to food production, processing, distribution, and preparation is critical when designing interventions to change behaviours at a local scale. Nevertheless, the global implications of our results are clear—foods marketed online as having immunity-boosting properties are, in general, healthier and better for global biodiversity and emissions than other foods.

Although digital media offer potential for delivery of public health interventions targeted at influencing voluntary behaviours (Webb et al. 2010; Cugelman et al. 2011; Young et al. 2019), there are major challenges in online information provision. Online information can be inaccurate, and is typically unverified (and sometimes even falsified) due to a lack of professional gatekeepers to monitor content (Scullard et al. 2010). False information is easily believed and socially amplified (Chung 2011). People’s access to the Internet, abilities to assess information accuracy and awareness of website credibility problems are variable and can be influenced by website design and socio-economic factors including digital information literacy (Sillence et al. 2007; Metzger et al. 2015). Whilst we ensured that all webpages reviewed were publicly available, not all people in each region we studied would have equal access to them. Additionally, our study did not evaluate the user experience (including the breadth of other information sources used by viewers), so we are unable to report whether the information on our reviewed webpages influenced people’s behaviours.

Many researchers fear that online health information currently being amplified could do more harm than good if people use inaccurate information to enact behaviour change. From a dietary perspective, this is because there is no evidence that individual food items offer any kind of immunity support on their own (Jayawardena et al. 2020; Venter et al. 2020). Scientific guidance on dietary changes to improve health and sustainability focuses on a balanced diet rich in fruits, vegetables and legumes (Willett et al. 2019). While websites promoting immunity-boosting foods are typically promoting consumption of individual foods in lieu of a total healthy diet, if the end outcome is a general increase in consumption of whole, plant-based foods, then this is a positive step to improved health and environmental sustainability. Our study was based on a sample of websites from a sample of countries. Lack of information verification meant it was not possible to determine whether website information provision was motivated by commercial interests or for public good. As an alternative to wholesale criticism of websites that are providing information, we suggest that health professionals and scientists find ways to increase the veracity of the information provided by working with, or providing communications for, such websites.

Public interest in the health and environmental impacts of food creates an opportunity to build an engaged and empowered community (Dean et al. 2016; Gardner et al. 2020). If engagement and understanding of the impacts of food choices can be converted to durable behaviour change, this is likely to have significant benefits not only for personal health but also global environmental sustainability. Obviously, it will take more than Internet information provision to enact real, large-scale changes to consumer behaviours that lead to durable positive outcomes for the environment and people’s health. Empowering the public to change eating habits and food consumption to more sustainable practices is complex and requires a combination of approaches to directly influence and encourage personal behaviours as well as creating supportive environments (e.g. through legislation) (de Ridder et al. 2017). However, online information should not be disregarded by those wishing to influence consumer behaviours. This is especially pertinent in times of crisis when people are more likely to seek out information (Procopio and Procopio 2007), and there is increased opportunity to engage with the public online and build awareness of optimal behaviours. This includes encouraging websites to promote whole diets, healthy dietary patterns, and increased serves of vegetables, which will contribute not only to a reduction in chronic health conditions but also simultaneously reduce the impacts of food consumption on the environment (Cugelman et al. 2011; Guthrie et al. 2015; Lehner et al. 2016; Ekwaru et al. 2017; Byerly et al. 2018).

## Supplementary Information

Below is the link to the electronic supplementary material.

Supplementary file1 (PDF 1310 kb)

Supplementary file2 (XLSX 187 kb)
