# Supplementary material for: Environmental and public health co-benefits of consumer switches to immunity-supporting food
Source: Ambio. 2022 Jan 25;51(7):1658–72. doi: 10.1007/s13280-021-01693-w (PMC8787970; doi:10.1007/s13280-021-01693-w)
Supplement: Supplementary file 1 — Supplementary file1 (PDF 1310 kb) [file 13280_2021_1693_MOESM1_ESM.pdf]

Ambio

Electronic Supplementary Material

Title: Environmental and public health co-benefits of consumer switches to immunity-supporting food

Authors: Ayesha I.T. Tulloch, Rachel R. Oh, Danielle Gallegos

Contents:

Table S1. Description of the 15 impact indicators, that are included in both model sets.

Table S2. Description of the predictor variables specified in each model across both model sets.

Figure S1. Relative representation of food group recommendations for (a) each study region and (b) each webpage type.

Figure S2. Results of PCA evaluating differences in composition and impacts of sets of food groups recommended on 150 websites across 6 countries offering advice about food items purported to have "immunity-boosting" properties, showing correlation biplots of individual webpages and variables for (a, c) 150 webpages and 23 food groups, and (b, d) 150 webpages and 12 indicators, grouped according to (a, b) six biogeographical regions and (c, d) six webpage types.

Figure S3. Relative risk of incidence or mortality from increasing the daily dose by one serving for each of 23 food groups, for six indicators of (a) all-cause mortality (ACM), (b) coronary heart disease, (c) colorectal cancer (CRC), (d) Diabetes, (e) Stroke, and (f) the average across all chronic diseases (CHD, CRC, diabetes, stroke).

Figure S4. Relative impact on environmental outcomes from increasing the daily dose by one serving for each of 23 food groups, for seven indicators of (a) land use, (b) greenhouse gas emissions (GHGs), (c) acidification, (d) eutrophication, (e) freshwater scarcity, (f) all terrestrial impacts (land use, GHGs, acidification) and (g) all aquatic impacts (acidification, eutrophication, freshwater scarcity).

Table S1. Description of the 15 impact indicators, that are included in both model sets.

Impact indicator group	Impact indicator	Description
Health	All-cause mortality (ACM)	The number of deaths from all causes.
	Coronary heart disease (CHD)	Relative risk of incidence or mortality from: total coronary heart disease or major coronary event, nonfatal myocardial infarction, any myocardial infarction, fatal myocardial infarction, incident ischaemic heart disease, fatal ischaemic heart disease, acute coronary syndrome)
	Colorectal cancer (CRC)	Relative risk of incidence or mortality from colorectal cancer.
	Type II diabetes (Diabetes)	Relative risk of incidence or mortality from type II diabetes.
	Stroke	Relative risk of incidence or mortality from: total stroke, ischaemic, haemorrhagic, intracerebral and subarachnoidal haemorrhage.
Environment	Land Use	A measure of how much land is occupied during food production, and includes those used to grow crops and/or livestock feed, to house animals.
	Greenhouse gas emissions (GHG)	A measure of GHG emissions from carbon dioxide, methane, and nitrous oxide, and reported in carbon dioxide equivalents. GHGs from activities include, but are not limited to, fertilizer production and application, manure management, and enteric fermentation.
	Acidification of soil (AP)	A measure of nutrient loading, and refers to the potential increase in acidity of an ecosystem. It is reported in SO_2 equivalents, and includes acidification potential from sulphur dioxide, nitrogen oxides, nitrous oxide, and ammonia, among others. It can result from

		activities such as fertilizer application, fuel combustion, and manure management.
	Eutrophication (EP)	A measure of nutrient runoff, and refers to the increase in nutrients entering an ecosystem. It is reported in PO_4 equivalents, and includes eutrophication potential from phosphate nitrogen oxides, ammonia, and ammonium, among others.
	Scarcity-weighted water use (Water Use)	A function that multiples water use by a constant that scales regionally based on water availability after demand from humans and aquatic ecosystems has been met.
Aggregate	Average relative health impact (RRAll)	The average of the relative impacts across all five health indicators.
	Average relative chronic disease impact (RRDisease)	The average of the relative impacts across four chronic disease indicators; coronary heart disease, colorectal cancer, diabetes and stroke.
	Average relative environmental impact (EnvAll)	The average of the relative impacts across all five environmental outcomes.
	Average relative terrestrial biodiversity impact (EnvTerr)	The average of the relative impacts across GHG emissions, land use and acidification of soil.
	Average relative aquatic biodiversity impact (EnvAq)	The average of the relative impacts for scarcity-weighted water use, acidification of soil and eutrophication.

Table S2. Description of the predictor variables specified in each model across both model sets.

Predictor	Description
Food group (binary)	Plant or animal
Impact indicator (categorical)	For each model, we included only one (out of 15 possible categories), choosing from: all-cause mortality; coronary heart disease; colorectal cancer; type II diabetes; stroke; land use; greenhouse gas emission; acidification of soil; eutrophication; water use; average relative health impacts; average relative chronic disease impact; average relative environmental impact; average relative terrestrial biodiversity impact; average relative aquatic biodiversity impact
Food group: Impact Indicator	Interaction term

(a) Countries

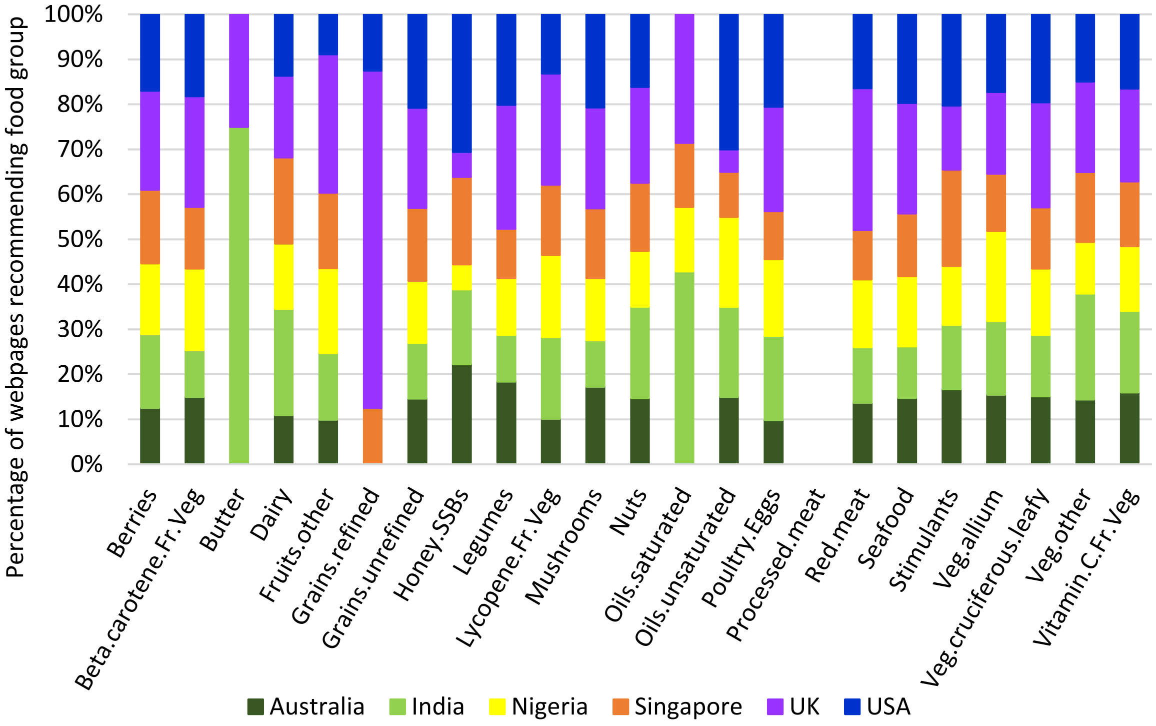

(b) Webpage types

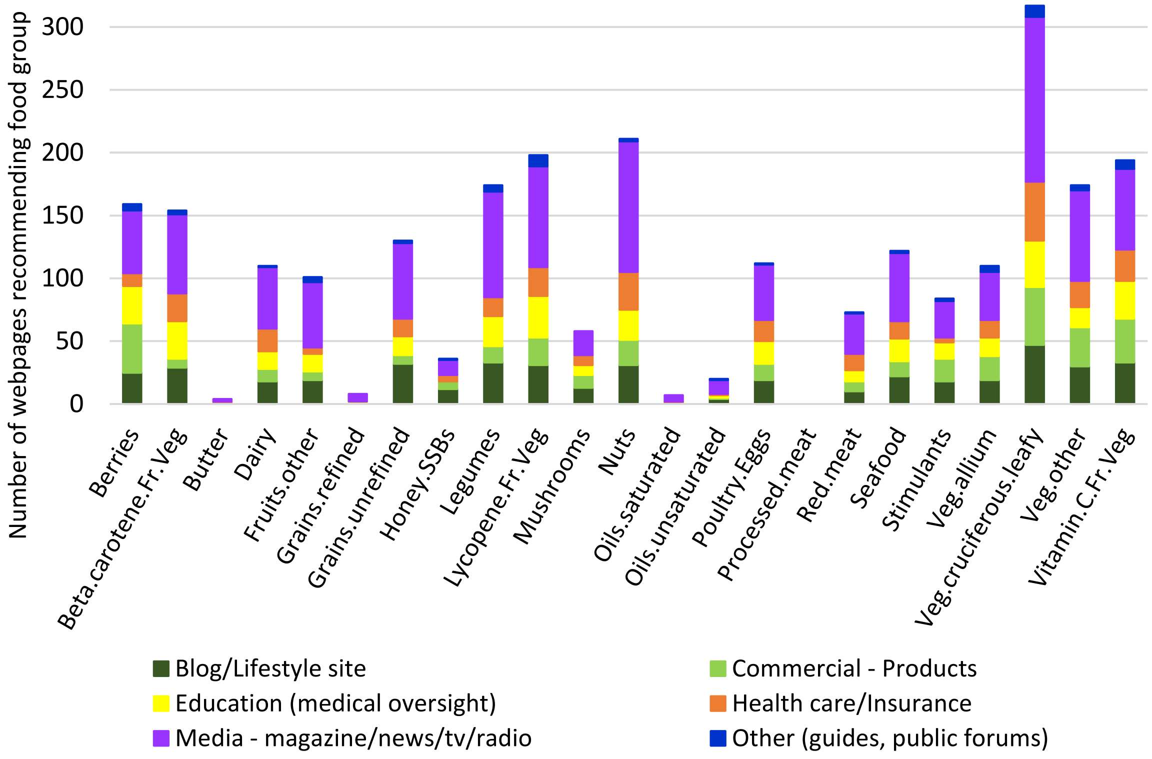

Figure S1. Relative representation of food group recommendations for (a) each study region and (b) each webpage type. Results for (a) are standardised to 100% so that the percentage represents, for each of the 23 food groups, what percentage of the total number of recommendations across all 150 websites were from a given country (e.g. 13% of the recommendations for berries across all the websites were from Australia). Results for (b) left unstandardized as sample sizes were unequal (e.g. there were 60 new media pages versus 16 health care/insurance pages).

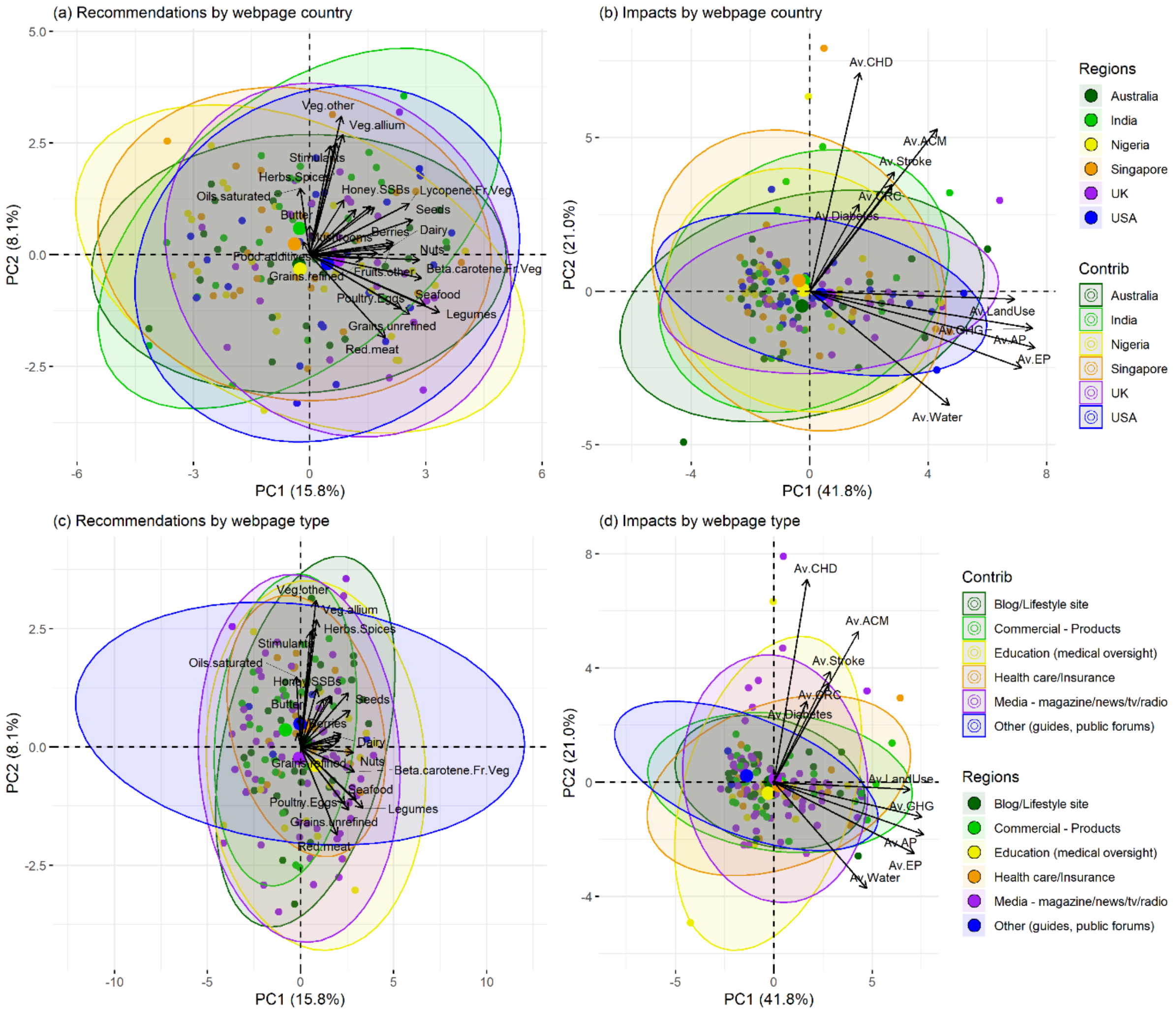

Figure S2. Results of PCA evaluating differences in composition and impacts of sets of food groups recommended on 150 websites across 6 countries offering advice about food items purported to have "immunity-boosting" properties, showing correlation biplots of individual webpages and variables for (a, c) 150 webpages and 23 food groups, and (b, d) 150 webpages and 12 indicators, grouped according to (a, b) six biogeographical regions and (c, d) six webpage types. The PCA indicated that there was no significant difference between regions in (a) the composition of sets of recommended food groups (ANOSIM R^2 = 0.06, P = 0.15), and (b) the average indicator value for each set of recommended foods (ANOSIM R^2 = 0.01, P = 0.20), and no significant difference between webpage types in (c) the composition of sets of recommended food groups (ANOSIM R^2 = 0.06, P = 0.95), and (d) the average indicator value for each set of recommended foods (ANOSIM R^2 = 0.02, P = 0.77).

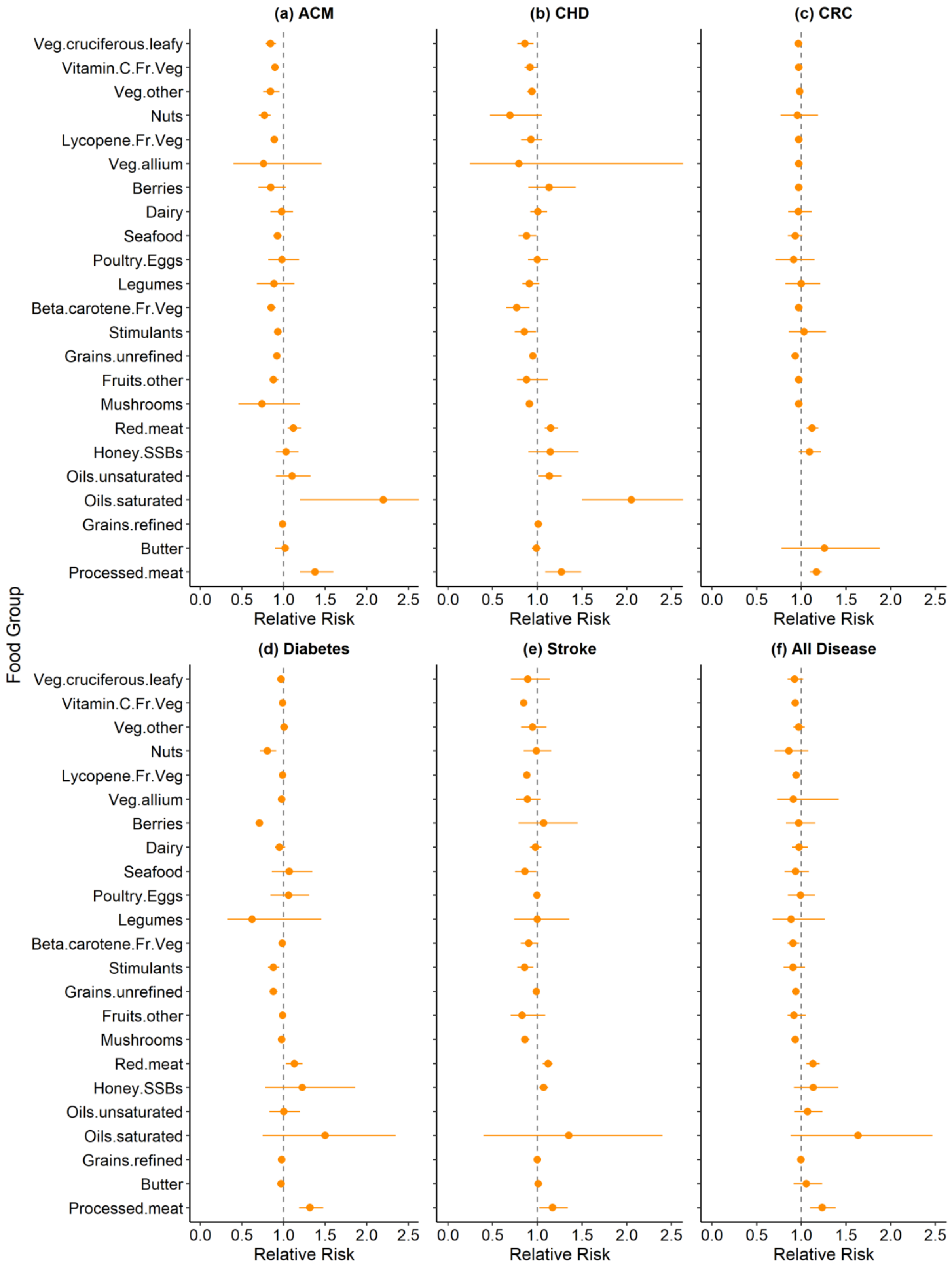

Figure S3. Relative risk of incidence or mortality from increasing the daily dose by one serving for each of 23 food groups, for six indicators of (a) all-cause mortality (ACM), (b) coronary heart disease, (c) colorectal cancer (CRC), (d) Diabetes, (e) Stroke, and (f) the average across all chronic diseases (CHD, CRC, diabetes, stroke).

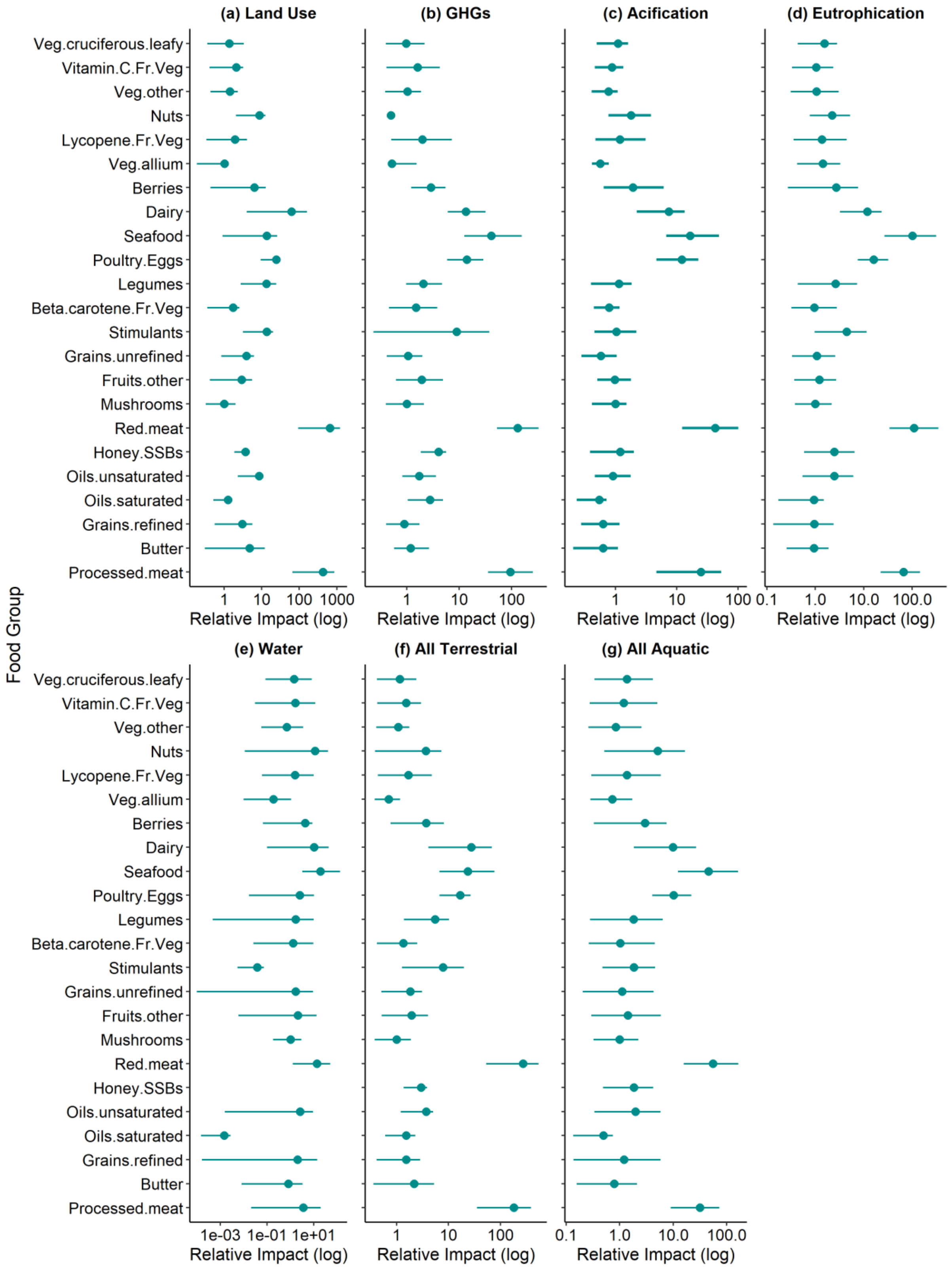

Figure S4. Relative impact on environmental outcomes from increasing the daily dose by one serving for each of 23 food groups, for seven indicators of (a) land use, (b) greenhouse gas emissions (GHGs), (c) acidification, (d) eutrophication, (e) freshwater scarcity, (f) all terrestrial impacts (land use, GHGs, acidification) and (g) all aquatic impacts (acidification, eutrophication, freshwater scarcity).
